# Three-Dimensional Printing in Breast Reconstruction: Current and Promising Applications

**DOI:** 10.3390/jcm13113278

**Published:** 2024-06-02

**Authors:** Horacio F. Mayer, Alejandro Coloccini, José F. Viñas

**Affiliations:** Plastic Surgery Department, Hospital Italiano de Buenos Aires, University of Buenos Aires Medical School, Hospital Italiano de Buenos Aires University Institute (IUHIBA), Buenos Aires C1053ABH, Argentina; alejandro.coloccini@hospitalitaliano.org.ar (A.C.); jose.vinas@hospitalitaliano.org.ar (J.F.V.)

**Keywords:** 3D imaging, 3D printing, 3D bioprinting, breast mold, scaffolds, biodegradable implants, flap shaping, tissue engineering, breast reconstruction

## Abstract

Three-dimensional (3D) printing is dramatically improving breast reconstruction by offering customized and precise interventions at various stages of the surgical process. In preoperative planning, 3D imaging techniques, such as computer-aided design, allow the creation of detailed breast models for surgical simulation, optimizing surgical outcomes and reducing complications. During surgery, 3D printing makes it possible to customize implants and precisely shape autologous tissue flaps with customized molds and scaffolds. This not only improves the aesthetic appearance, but also conforms to the patient’s natural anatomy. In addition, 3D printed scaffolds facilitate tissue engineering, potentially favoring the development and integration of autologous adipose tissue, thus avoiding implant-related complications. Postoperatively, 3D imaging allows an accurate assessment of breast volume and symmetry, which is crucial in assessing the success of reconstruction. The technology is also a key educational tool, enhancing surgeon training through realistic anatomical models and surgical simulations. As the field evolves, the integration of 3D printing with emerging technologies such as biodegradable materials and advanced imaging promises to further refine breast reconstruction techniques and outcomes. This study aims to explore the various applications of 3D printing in breast reconstruction, addressing current challenges and future opportunities.

## 1. Introduction

According to the American Society of Plastic Surgeons, 151,641 breast reconstructions were performed in the United States in 2022 [1]. Advances in early detection of breast cancer, combined with improved management and treatment, resulted in a reduction in mortality rates, despite a slight increase in global incidence. The growing numbers of long-term survival of patients diagnosed with breast cancer have allowed more attention on quality of life after treatment [2,3].

An important aspect of quality of life post-treatment is breast reconstruction, which aims to restore the appearance and symmetry of the breasts [4]. This reconstruction can be performed with implants or with autologous tissue. Although implant-based reconstruction is the most widely used and is associated with shorter surgical times, reconstructions with autologous tissue provide a more natural appearance of the breast, as well as a higher quality of life [5,6]. However, existing reconstructive methods may have limitations in achieving optimal results, which has prompted the exploration of different alternatives, such as three-dimensional (3D) printing. This technology has emerged as a promising tool that offers possible solutions to improve aesthetic results and increase patient satisfaction, achieving a more natural and accurate reconstructive result through its great versatility [7].

This study aims to review the current and promising applications of 3D printing in breast reconstruction.

## 2. Current Technologies of 3D Bioprinting

Computer-aided design and manufacturing (CAD/CAM) techniques, commonly known as 3D printing, bring together those processes that allow the manufacture of solid objects by successive printing of thin layers of different types of materials. Their origins are related to the patenting in 1986 by Charles Hull of the first commercial 3D printing technology by stereolithography [8]. This method consists of a CAD/CAM technique where 3D objects are converted into triangles representing coordinates encoded in binary or ASCII format in order for the 3D printer to precisely place each part of the object in space. This is materialized by the solidification (polymerization) of a liquid material (photosensitive liquid polymer) contained in a bath when exposed to ultraviolet radiation emitted by a laser [9].

Other 3D bioprinting techniques such as selective laser sintering, fused deposition modeling and inkjet printing were also developed from this innovative technology [10].

## 3. Preoperative Applications

### 3.1. Surgical Planning

**Figure 1:**
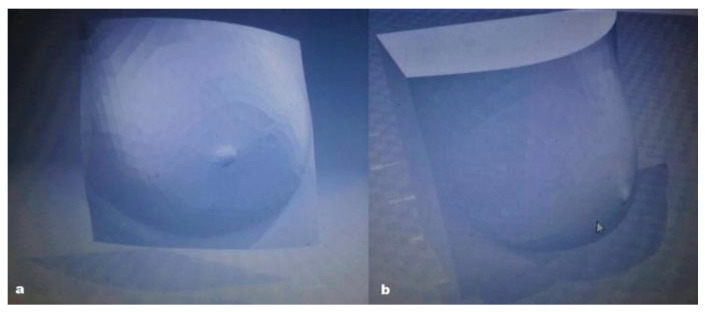
Editing and design of the 3D breast model: (**a**) frontal view; (**b**) oblique view (reproduced with permission).

A cornerstone of the 3D bioprinting process is the acquisition of 3D surface images of the breast. These images are used to create detailed digital models (Figure 1), which form the basis for the elaboration of 3D bioimpressions. A great advantage of these 3D models is that they can be modified, allowing adaptation to the specific needs of each patient to achieve the desired result. In addition, it enables the surgeon to carry out a thorough analysis of the anatomy and characteristics of the breast tissue. It even gives the surgeon the opportunity to try different surgical approaches to improve the surgical plan and obtain optimal results [11].

Traditional imaging methods that can be used to develop 3D models include computed tomography (CT) and magnetic resonance imaging (MRI). Although both are suitable for estimating breast volume, they are inadequate for assessing breast morphology. This is because the patient’s position (supine or prone, respectively) during these studies alters its natural shape in the standing position [12].

**Figure 2:**
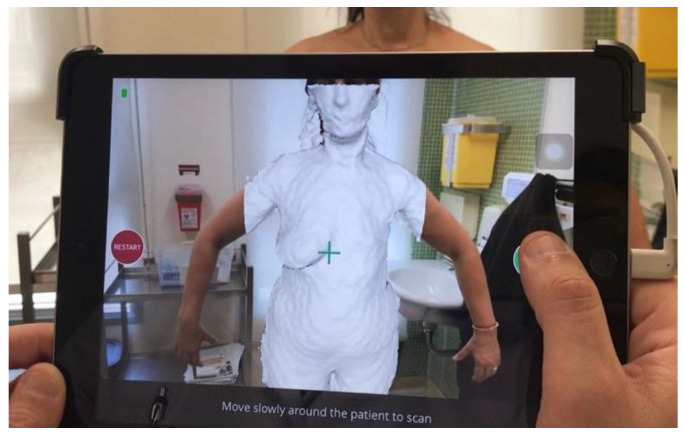
Use of simulator software to obtain images of the 3D breast surface with the patient in a standing position (reproduced with permission).

An alternative to achieve a reproduction of the breast morphology in this position is 3D body surface imaging. One technology that allows the creation of 3D models is the laser scanner. This device emits beams of light onto the breast surface in a repetitive pattern, calculating the distance to the surface by detecting the orientation of its reflection. Another technique used for this purpose is stereophotogrammetry, which constructs the 3D model using cameras placed at different angles [13]. Although these technologies have been used in several studies, they have some disadvantages [14,15,16,17]. These include their high cost, the need for a specific physical space and the requirement of specialized training for their use, which hinders their practical application. However, an interesting solution to these drawbacks is the adoption of web-based 3D simulation software (Virtual Aesthetics, Crisalix, Lausanne, Switzerland) designed specifically for the field of plastic surgery, requiring only a portable 3D sensor connected to a tablet (Figure 2) [18,19].

As previously mentioned, it is feasible to print 3D biomodels from 3D virtual models, which facilitates the surgeon’s interaction with the patient’s breast anatomy. These not only offer visual and tactile information, but also make it possible to reproduce the surgical procedure prior to its execution, which reduces the possibility of complications and guarantees more predictable results. In addition, their use allows a more efficient management of surgical resources and would contribute to shortening surgical times [12,20,21].

### 3.2. Three-Dimensional Breast Volume Measurement

Measurement of breast volume is essential to achieve satisfactory results during breast reconstruction [22,23]. At present, the lack of a simple and replicable method to calculate it limits its applicability in practice. Among the various techniques available, manual anthropometric measurements, the Archimedes method (based on water displacement), the Grossman–Roudner device (which uses conical discs inside which the breast is placed), mammography, CT, MRI and 3D surface imaging can be highlighted [24,25].

While the ability to perform adequate manual measurements is essential for surgeons, 3D measurements may be useful for those less experienced in this type of surgery, considering its advantages in terms of cost, applicability, safety, being a non-invasive method and avoiding radiation exposure to patients. This measurement process begins with the scanning of the breast surface contour, to which a simulated representation of the chest wall is then incorporated. The resulting volume of this 3D figure is used as a measure of breast volume. However, it is important to keep in mind the limitations of this technology, which are detailed in Table 1 [26,27,28].

Breast volume measurement offers a quantitative guide for breast reconstructions, whether implant-based or autologous [29].

### 3.3. Planning Prosthetic Reconstruction

Nowadays, immediate reconstruction with implants is the main method after a mastectomy. An increasing number of surgeons prefer the one-stage approach, using implants directly, to the two-stage approach with tissue expanders [5,30].

Selecting an adequate implant for breast reconstruction can be difficult due to the numerous options available. In doing so, it is important to take into account factors such as tissue changes due to oncologic treatments and patient preferences [31,32]. In view of the limitations of volumetric measurements of the breast, surgeons often use linear measurements (height, width and projection) along with their clinical experience to choose implants [12,33,34]. In part, this is because manual breast volume measurements often involve complicated formulas or are too time-consuming, which can be impractical [35,36].

Several studies have been performed using 3D scanning to predict prosthetic size from preoperative breast volumetric measurements in immediate one-stage reconstructions [32,37,38,39,40]. Nonetheless, various factors such as breast asymmetries, tumor staging, oncologic resection and patient preferences make this determination difficult [39,40].

Regarding two-stage reconstruction with tissue expanders, more promising results have been obtained through the use of 3D imaging. It has been observed that volumetric data from the contralateral side can serve as a reference for selecting expander size, final expansion volume and final implant size/shape [41,42,43]. It also helps to determine the type of symmetrization surgery needed on the contralateral breast, whether it is a mastopexy, augmentation or reduction mastoplasty [44].

Although improvements in breast symmetry were reported with the use of this technology, more multicenter randomized controlled trials are needed to validate these findings.

### 3.4. Planning Autologous Reconstruction

Among the alternatives for autologous breast reconstruction, the deep inferior epigastric artery perforator (DIEP) flap is still considered the gold standard [45,46]. In this procedure, abdominal tissues, which include subcutaneous fat and skin from the lower abdomen, are transferred as a vascularized free flap to reconstruct the breast. This not only provides a texture similar to that of the original breast tissue, but also prevents complications related to breast implants, lasts over time and integrates harmoniously with the patient’s body [47,48].

Flap survival is closely linked to the correct identification of the dominant perforator [49,50]. Usually, these vessels present a sinuous course as they run through the rectus abdominis muscle, which increases the risk of unwanted vascular injury during intramuscular dissection.

Though angiotomography with 3D reconstruction is the standard for visualizing perforators, its accuracy in locating them along their intramuscular subfascial course is limited [51,52,53]. By not considering variations in depth along their trajectory, it makes it difficult to transpose these location points to the abdominal wall during preoperative marking, making the procedure subject to possible inaccuracies [54].

Recently, technological advances have facilitated the 3D printing of real-size templates using angiotomographic data of the anatomy of the perforator vessels [54,55,56,57,58,59]. This contributes to preoperative surgical planning and, by being able to generate sterilizable models, serves as intraoperative guidance during dissection of the intramuscular course of the perforators. Despite the above benefits, the costs associated with the purchase of 3D printers and materials, although gradually decreasing, along with the time required to print 3D models, are some of the limitations that hinder their widespread adoption nowadays [60].

**Figure 3:**
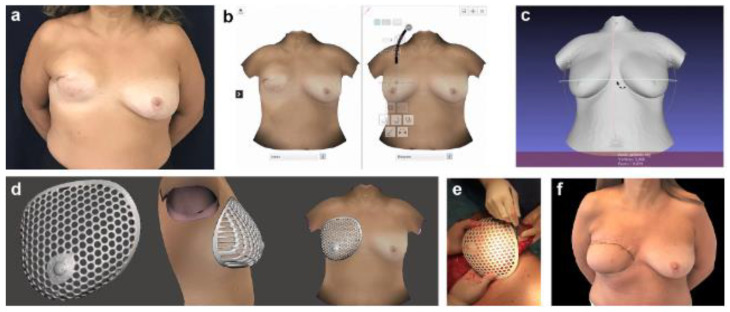
(**a**) Anterior preoperative view of a 59-year-old patient reconstructed with an abdominal-based flap and the use of breast molds to optimize results; (**b**) the remaining contralateral breast is rendered and mirrored; (**c**) the rendered breast image is exported for edition; (**d**) design of the biomodel; (**e**) the biomodel is used to shaping the flap intraoperatively; and (**f**) anterior postoperative view of the same patient at 45 days (reproduced with permission).

**Figure 4:**
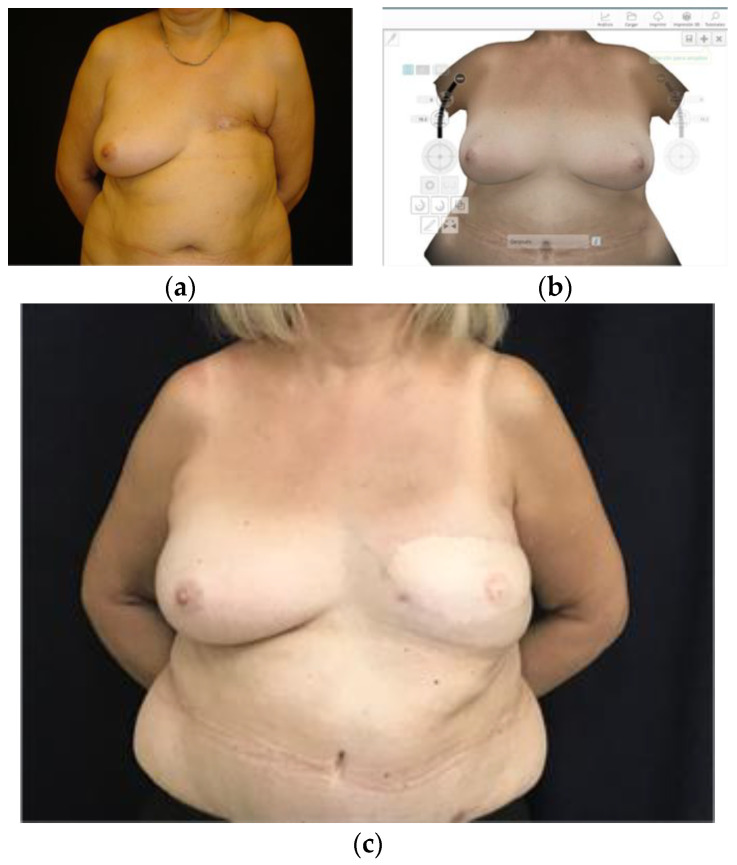
A 68-year-old patient in whom a breast biomodel was used to optimize breast reconstruction results in terms of shape, volume and symmetry. (**a**) Preoperative frontal view; (**b**) the remaining contralateral breast is rendered and mirrored to create a customized biomodel; and (**c**) postoperative frontal view at one year.

It is worth mentioning that preoperative 3D images have also been successfully used to estimate the necessary volume of flaps in autologous breast reconstructions, and 3D printed breast molds have even been produced to assist the surgeon in defining the shape and size of the breast (Figure 3 and Figure 4) [16,61]. However, it is relevant to note that the sample sizes in the studies conducted have been limited, highlighting the need for further research to strongly support their clinical applications.

## 4. Intraoperative Applications

### 4.1. Implants Customization

The goal of breast reconstruction is to restore the shape, appearance, symmetry and size of the breasts after mastectomy or lumpectomy [62]. However, despite the wide variety of standardized breast implants available on the market, their optimal adaptation to each individual case is not guaranteed, which can lead to aesthetic problems such as asymmetry [63,64]. The manufacture of custom-made silicone breast implants using 3D printing could offer an innovative solution to this problem [20,63,65]. Even though to date we have not found studies evaluating their clinical application, the successful use of silicone elastomer implants in cases of pectus excavatum [66,67] and Poland’s syndrome [68] for chest wall reconstruction has been described.

Meanwhile, a study has been conducted in which porous, biodegradable polycaprolactone breast implants were fabricated using custom 3D printing, although further research is needed to support their clinical application [69].

### 4.2. Flap Modeling with Scaffolds

Three-dimensional printing of customized breast molds simplifies flap modeling in autologous breast reconstructions, which is beneficial considering the extensive learning curve required given the high complexity of the procedure. This approach involves adapting the autologous tissue within a mold to fit the desired breast dimensions in order to optimize the results [14,15,16,19,70].

This methodology is adaptable to various situations. In immediate unilateral breast reconstructions, the unaffected breast is used as a reference point for the fabrication of a 3D mold in patients satisfied with its shape and size. In immediate bilateral reconstructions, the most aesthetically pleasing breast is chosen as the model prior to mastectomy.

In some cases, such as delayed bilateral reconstructions, preoperative 3D images may not be available. Under such conditions, a virtual representation of the breast can be created that is tailored to the specific needs of the patient.

Another particular circumstance represents the need for a reduction mastoplasty or mastopexy for breast ptosis of the reference breast. Tomita et al. [71] described the placement of a tissue expander on the oncologic side and mastopexy of the contralateral breast during the initial surgery. Between four and six months postoperatively, they created a 3D printed mold using the mastopexy-corrected breast as a guide. This mold was used in a second procedure to shape the DIEP flap. The time interval between the two surgeries was crucial, as it enabled the postoperative morphologic changes of the mastopexy.

In another study [72], anthropometric data from 15 patients who underwent DIEP flap breast reconstruction were used to develop 10 molds known as “DIEP sizers”. A patient whose reconstructed breast matched the average parameters of the group was selected, serving as a reference model for the size and shape of the molds. The sizer was selected according to each patient’s physical characteristics, facilitating the positioning and shaping of the flap during surgery. The authors highlighted that the creation of different reusable and resterilizable DIEP sizers would allow their applicability in a large number of patients, reducing production time and costs. It would also benefit those cases lacking a suitable contralateral breast as a reference.

### 4.3. Tissue Engineering Based on 3D Printed Scaffolds

An alternative to implant-based breast reconstruction is autologous adipose tissue grafting. Although lipografts have many advantages, such as biocompatibility, simplicity of the procedure, natural cosmetic results, low cost and reduced complication rates, a 30% to 40% volume loss has been observed after lipotransfer [73,74,75,76]. Tissue engineering using 3D printed scaffolds employs a support structure to facilitate cell development, thus mimicking the function of the extracellular matrix under normal conditions [77]. As a result, it could provide structural support tailored to individual patient needs, prevent lipograft resorption and promote regeneration. While the exact mechanism behind these two processes is not fully understood, it is likely that they are related to vascularization [78].

Tissue regeneration is directly influenced by the mechanical and chemical characteristics of the scaffold used, as well as its porosity [78,79,80].

#### 4.3.1. Features of the Scaffold Structure

The mechanical characteristics of the scaffold should resemble those of breast tissue. Excessive stiffness can trigger the formation of scar tissue through a marked inflammatory process, while a structure that is too flexible risks collapsing, thus hindering tissue survival and regeneration [81].

A determinant in the performance of 3D scaffolds is porosity as it allows cell migration and angiogenesis necessary for the development of new tissues [82]. On the other hand, the biodegradation rate of the scaffold is also fundamental, as it must be maintained long enough for the formation of new tissue, but it must also allow its replacement by the extracellular matrix [83].

#### 4.3.2. Scaffolding Biomaterials

The biomaterial that forms the scaffold can be a biological or synthetic polymer. Biological polymers stand out for their high biocompatibility as they possess molecular properties analogous to the extracellular matrix. However, they have limited mechanical strength and tend to degrade rapidly in the presence of body fluids [79,83]. Within this group, hydrogels (collagen, gelatin, fibrin, hyaluronic acid, chitosan, alginate, among others) stand out for their potential in tissue engineering. Hydrogels are a porous network that can retain significant amounts of water or biological fluids. They enable the inclusion of living cells in scaffolds and can even provide growth factors [84].

On the other hand, synthetic polymers offer the ability to largely control their mechanical, degradative and hydrophobic properties. Compared to natural polymers, they provide greater mechanical stability, and it is simpler to add growth factors and extracellular matrix components to them. Their main disadvantage lies in their limited biocompatibility due to the absence of peptides and binding sites, requiring chemical modifications on the surface to enhance tissue regeneration [80,83]. The wide variety of available synthetic polymers such as polycaprolactone (PCL), polylactic acid (PLA) and poly(lactic-co-glycolic acid) (PLGA), together with the ability to integrate bioinks with cells, gives rise to new opportunities for innovation in the field of breast reconstruction.

The current challenge in the field of breast reconstruction is to prevent resorption and stimulate adipogenesis in moderate to large volumes of lipotransfer [78]. Encouraging results have been reported with delayed lipotransfers after breast implantation of scaffolds with a combined structure: an outer layer providing biomechanical support and an inner layer guiding tissue proliferation [85,86]. Adipose tissue-derived stem cells can be easily isolated with lipoaspiration, are more resistant to poor vascularization than adipocytes and are multipotent, making them ideal for angiogenesis and adipogenesis [80,83,87]. However, their use carries the risk of contributing to neoplastic recurrences in the breast [88]. To date, there are no studies supporting their safety in long-term clinical practice [64].

Two different clinical trials are currently being carried out with resorbable scaffolds in which a pedicled fat flap is placed [89,90]. This approach could be a valid option for patients requiring radiotherapy, avoiding the breast alterations it produces, and without the risk of neoplastic recurrences associated with the reconstructive method.

## 5. Postoperative Applications

### Objective Assessment of Outcome

Postoperative outcomes of the reconstructed breast are determined by factors such as shape, size and symmetry [91]. Three-dimensional imaging is a validated, accurate method of assessing breast dimensions compared to in-person measurements and has been used for evaluating implant- and flap-based breast reconstruction outcomes in several studies [41,91,92,93,94,95,96].

Pre- and postoperative 3D photographs are analyzed to obtain and compare objective measurements of total breast volume, breast base diameter, submammary sulcus height, breast mound projection (anteroposterior projection) and surface curvature, among others, which most commercially available 3D imaging software is capable of measuring [41,93]. In addition, 3D imaging allows an objective assessment of the symmetry between the reconstructed and contralateral breast [41,94].

Three-dimensional breast data greatly enhance our ability to assess surgical outcomes. Since 3D photography documents the true changes in shape and tissue distribution that occur over time, this technology helps to identify the pitfalls and success of each procedure [41,91,93,95]. Thus, long-term postoperative breast volume changes can be investigated by 3D imaging, for example, after autologous breast reconstruction with a free perforator flap [96]. The use of three-dimensional imaging in implant-based reconstruction has also been described to determine the volumetric differences between the expanded and contralateral breast, which is beneficial as a method to assess tissue expansion and the need for symmetry or revision procedures and to critically analyze the final reconstructive outcome [41].

In terms of patient satisfaction, several tools exist for assessing satisfaction with the long-term aesthetic outcome after breast reconstruction surgery. In this regard, 3D imaging has been used as a more objective approach to assess the aesthetic outcome in terms of volume and shape symmetry, although one study has suggested that this does not translate directly into patient-reported satisfaction [97].

## 6. Three-Dimensional Printing as an Educational Tool

Three-dimensional printing can enable a deeper understanding of human anatomy, traditionally gained from textbook drawings and years of surgical experience in performing complex dissections, so rapid prototyping is an evolving technology that has the potential to revolutionize medical education [20]. Mehta et al. applied 3D printing to autologous reconstructive breast surgery by creating a patient-specific model that helped teach DIEP flap breast reconstruction to trainee surgeons who used the model preoperatively and postoperatively to visualize the intramuscular path of the deep inferior epigastric perforator vessels [56]. Papavasiliou et al. developed a 3D printed chest wall as an adjunct to the current chicken thigh model that mimics the anastomosis performed during DIEP breast reconstruction, representing a simple and cost-effective enhancement that provides a significantly more realistic resemblance to a clinical situation than the original model [98]. Lastly, Lim et al. reported the use of a novel simulator with different breast volumes and ptosis grades in a single model for teaching marking in oncoplastic surgery [99]. In this regard, the future of plastic surgery education is exciting because of the ability to take a two-dimensional (2D) image and bring it to life with a full-scale model [20,63].

To obtain a complete understanding of the various uses of 3D printing in each phase of the breast reconstruction process, a detailed summary is presented in Table 2.

## 7. Current Challenges and Future Directions

The future of 3D-printing-assisted breast reconstruction promises to be revolutionary. Although still in its early stages, everything suggests progress towards regeneration of functional breast tissue. So far, mature, hormone-sensitive breast tissue has been successfully developed from primary human breast epithelial cells seeded in 3D-printing-based hydrogels [100]. Still, the current challenge remains the risk of local recurrence of breast cancer [88], limiting its application in humans.

Three-dimensional printing has been essential for the advancement in the manufacture of tissues and customized implants. However, the next phase will be four-dimensional (4D) printing, which makes it possible to create structures capable of adapting to external stimuli and releasing chemotherapeutic drugs or antibiotics in a controlled manner [101]. One limitation of these structures is their reduced ability to load drugs. To overcome this problem, Dang et al. combined 3D printing with the porogen leaching technique, creating pores of different sizes [102]. They experimented with three drugs (doxorubicin, paclitaxel and cefazolin), finding that the smaller pores allowed the structures to load and release the drugs in an effective and controlled mode for extended periods of time.

For its part, artificial intelligence can enhance both 3D and 4D printing by aiding decision making during preoperative planning, simplifying material selection and streamlining production processes [103,104].

As science moves toward the convergence of 3D and 4D printing with artificial intelligence, it will be imperative to maintain a constant focus on patient safety throughout the entire breast reconstruction process.

## 8. Conclusions

Breast reconstruction has advanced significantly with 3D printing, improving both aesthetic results and patient satisfaction. Applications of this technology include preoperative planning, creating patient-specific surgical models; intraoperative uses, such as customizing implants and molds for more accurate results; and postoperative uses, through objective evaluations of surgical results with 3D images of breast volume and symmetry. It also serves as an educational tool, enabling the manufacture of more realistic anatomical models for surgical training. Despite these advantages, more prospective clinical randomized trials are needed to validate its widespread use. Looking forward, the integration of 4D printing and artificial intelligence promises even more personalized and dynamic treatments, in which patient safety will remain a key issue.

## Figures and Tables

**Table 1:** Limiting factors of 3D surface imaging scanning for breast volume measurement.

Limiting Factor	Description
Breast base	It does not detect the boundary between the breast and the chest wall, so it is necessary to simulate the latter with software from the surrounding chest wall.
High BMI ^1^	Difficult to precisely define the lateral border of the breast.
Severe breast ptosis	Difficult to locate the submammary fold.
Movement and skin color	Postural variations, respiratory movements during scanning and patient skin tone may affect measurements.

^1^ BMI: body mass index.

**Table 2:** Applications of 3D printing in breast reconstruction.

Applications	Description
Preoperative Applications	
Surgical Planning	Creation of 3D ^1^ digital models for preoperative analysis. Modification of models to adapt to patient-specific needs. Simulation of surgical procedures to enhance planning.
2. Three-Dimensional Breast Volume Measurement	Surface scanning of the breast for preoperative volume calculation.
3. Planning Prosthetic Reconstruction	Utilization of 3D scanning to select appropriate implant size.
4. Planning Autologous Reconstruction	Creation of 3D templates from the anatomy of perforating vessels.
Intraoperative Applications	
Implants Customization	Customized manufacturing of silicone breast implants through 3D printing.
2. Flap Modeling with Scaffolds	Flap modeling using customized molds.
3. Tissue Engineering with 3D Printed Scaffolds	Application of 3D printed scaffolds for autologous regeneration of adipose tissue.
Postoperative Applications	
Objective Assessment of Outcome	Three-dimensional image analysis to measure breast volume, shape and symmetry.
Educational Applications	
Patient-Specific Surgical Models	Preoperative and postoperative visualization of vascular paths for microsurgery training.
2. Enhanced Anatomical Models	Improved realism compared to traditional models for procedural training.
3. Variable Anatomy Simulation Models	Teaching surgical techniques with variable anatomical conditions.

^1^ 3D: Three-dimensional.
